# Gas Chromatography-Mass Spectrometry (GC-MS) Metabolites Profiling and Biological Activities of Various *Capsicum annum cultivars*

**DOI:** 10.3390/plants11081022

**Published:** 2022-04-09

**Authors:** Rizwan Ahmad, Aljawharah Alqathama, Mohammed Aldholmi, Muhammad Riaz, Ashraf N. Abdalla, Ahmed Mostafa, Hamdi M. Al-Said, Abdulmalik M. Alqarni, Riaz Ullah, Sami S. Asgher, Mohd Amir, Heba Shaaban, Wasim Ahmad

**Affiliations:** 1 Department of Natural Products and Alternative Medicine, College of Clinical Pharmacy, Imam Abdulrahman Bin Faisal University, Dammam 31441, Saudi Arabia; mjaldholami@iau.edu.sa (M.A.); matahmad@iau.edu.sa (M.A.); 2 Department of Pharmacognosy, Faculty of Pharmacy, Umm Al-Qura University, Makkah 21955, Saudi Arabia; aaqathama@uqu.edu.sa; 3 Department of Pharmacy, Shaheed Benazir Bhutto University, Sheringal 18050, Pakistan; pharmariaz@gmail.com; 4 Department of Pharmacology and Toxicology, Faculty of Pharmacy, Umm Al-Qura University, Makkah 21955, Saudi Arabia; anabdrabo@uqu.edu.sa; 5 Department of Pharmacology and Toxicology, Medicinal and Aromatic Plants Research Institute, National Center for Research, Khartoum 2404, Sudan; 6 Department of Pharmaceutical Chemistry, College of Clinical Pharmacy, Imam Abdulrahman Bin Faisal University, Dammam 31441, Saudi Arabia; ammostafa@iau.edu.sa (A.M.); amalqarni@iau.edu.sa (A.M.A.); hsmohammed@iau.edu.sa (H.S.); 7 Department of Microbiology, College of Medicine, Umm Al-Qura University, Makkah 21955, Saudi Arabia; hmibrahim@uqu.edu.sa (H.M.A.-S.); ssasgher@uqu.edu.sa (S.S.A.); 8 Department of Pharmacognosy (MAPPRC), College of Pharmacy, King Saud University, Riyadh 11451, Saudi Arabia; rullah@ksu.edu.sa; 9 Department of Pharmacy, Mohammed Al-Mana College for Medical Sciences, Dammam 34222, Saudi Arabia; wasimahmadansari@yahoo.com

**Keywords:** capsicum, cytotoxicity, GCMS, antimicrobial, PCA, K-mean

## Abstract

This study evaluates the quality variation for twenty-seven capsicum fruit (CF) samples, in terms of their volatile oil composition and biological activities. The GCMS analysis revealed the presence of seventy one chemical compounds from different chemical classes with an average (%) composition of: 26.13 (alcohols) > 18.82 (hydrocarbons) > 14.97 (esters) > 3.08 (ketones) > 1.14 (others) > 1.07 (acids) > 0.72 (sugar) > 0.42 (aldehydes) > 0.15 (amino compounds). Alcohols and hydrocarbons were the most abundant in these CF samples with 1-Decanol, 2-octyl- and docosanoic acid, docosyl ester as the major components, respectively. The % inhibition in cytotoxicity assays was observed in the range of 9–47 (MCF7) and 4–41 (HCT116) whereas, the zone of inhibition (mm) for the antimicrobial activity was found to be 0.0–17 (*P. aeruginosa*) > 0.0–13 (*E. coli* and *S. aureus*). Moreover, the samples with the largest zone of inhibition in the agar-well-diffusion method (C16, C19, and C26) upon further evaluation presented the least MIC and MBC values against *P. aeruginosa* with an MIC and MBC (µg/mL) of 6.3 and 12.5, respectively. The outcome for GCMS and biological activities were further supported by statistical tools of PCA and K-mean cluster analysis which confirmed the C16 CF sample with the best activity followed by C5, C13 (the best cytotoxic), and C19, C26 (the best antimicrobial). The statistical analysis exhibited a high Chi-square value of 5931.68 (GCMS) and 32.19 (biological activities) with *p* = 0.00 for KMO and Bartlett’s Test of Sphericity. The 27-CF samples were effectively distinguished based on quality variation, and the C16 CF sample exhibited significant potential for further study.

## 1. Introduction

*Capsicum annuum*, commonly known as chili or pepper, is a flowering plant of the Solanaceae family that has been used worldwide as a spice and an ingredient in food and medicinal products [1]. It is largely used in the food and beverage industry as a coloring and flavoring agent. There are many varieties of chili with a wide range of sizes, colors, and shapes of fruits, as well as different levels of pungency (hot or sweet). It was estimated that approximately 19 million tons of chili were produced in 2001 by different countries on a cultivation area of around 1.5 million hectares [2]. The production increased to 38 million tons of fresh chili and 4.2 million tons of dry chili in 2019 [3]. Asia is responsible for the production of greater than 60% of the global production, with China and India being the largest producers of fresh and dry chili, respectively [4]. Chili contains various phytochemicals, including capsaicinoids and carotenoids. The former are alkaloids that are responsible for the characteristic pungency of hot chili while the latter are the primary pigments that give chili its distinctive color [5]. Capsaicin and di-hydrocapsaicin collectively represent over 80% of capsaicinoids in chili while the other derivatives such as nordihydrocapsaicin, homodihydrocapsaicin, homocapsaicin, norcapsaicin, nornorcapsaicin, nornornorcapsaicin, and nonivamide are present in very small quantities [6]. The pigments responsible for the color consist of xanthophylls such as capsanthin, zeaxanthin, cryptoxanthin, capsorubin, and lutein along with other carotenoids such as alpha and beta-carotenes [7]. Other chemical components present in chili involve volatile molecules, fatty acids, phenolics, vitamins (C and E), and minerals [4]. Several studies have reported a wide range of biological activities for chili extracts including antimicrobial activity against fungi and bacteria (both gram-positive and gram-negative bacteria), although the studies on the compounds responsible for these activities are limited [8,9,10,11]. In contrast, the antioxidant capacity of chili is believed to be mostly attributable to carotenoids and polyphenols as well as nutrients such as vitamins [12]. Moreover, chili extracts and capsaicin have shown anti-proliferative activities in in vitro and in vivo studies against several human cancer cell lines, including lung, breast, gastric, and prostate cancer cell lines [13,14,15].

The number of capsicum cultivars is available in the market, which may compromise the quality of capsicum in terms of variation of active ingredients and the quantities present in the samples. Capsaicinoids and carotenoids are the main quality parameters that are indicative of the pungency and color of chili [16]. Pungency is typically presented as Scoville Heat Units (SHU), where 16 SHU correspond to 1 ppm of capsaicin and capsaicin derivatives. Similarly, the color of chili is measured using the American Spice Trade Association (ASTA) method based on the absorbance of chili acetone extract at 460 nm [17]. Another critical quality parameter is the aroma of chili represented by the volatile fraction of the fruits [18]. The technique of choice for the analysis of volatile compounds is gas chromatography-mass spectrometry (GC-MS). This technique has been used to measure the changes in volatile profiles during different stages of ripeness in several studies [17,19,20,21]. The volatile fraction has been shown to be composed of different chemical classes, including alcohols, hydrocarbons, ketones, aldehydes, fatty acids, pyrazines, esters, monoterpenes, and sesquiterpenes [22]. However, there is a lack of research to measure the differences between volatile profiles from different cultivars and assess the correlation between the volatile profiles. This study will investigate the comprehensive volatile profile for all the market-available capsicum fruit (CF) samples and its potential role in the biological activities of cytotoxicity and antimicrobial activity.

## 2. Results

### 2.1. GC-MS

Seventy one volatile chemical compounds from different chemical classes were observed during GCMS analysis. The % average occurrence of these chemical classes may be ordered as: 26.13 (alcohols) > 18.82 (hydrocarbons) > 14.97 (esters) > 3.08 (ketones) > 1.14 (others) > 1.07 (acids) > 0.72 (sugar) > 0.42 (aldehydes) > 0.15 (amino compounds). The predominant among the chemical classes were alcohols (26.13%) and hydrocarbons (18.82%) with 2-(Octadecyloxy)-ethanol (0.05–16.9%) and docosanoic acid, docosyl ester (0.43–48.85%) as the major components, respectively shown in Table S1. The representative chromatograms for some CF samples are shown in Figure S1.

### 2.2. Cytotoxicity

The general screening of 27-CF samples for cytotoxicity revealed a mean (±SD) with a range for activities against the cell lines as: MCF7 29.14 ± 10.03 (9.0–47) and HCT116 19.33 ± 9.8 (3.0–41). (Table 1) The CF samples with the highest % inhibition against MCF7 and HCT116 were C5 (42 and 37), C13 (45 and 40), and C16 (47 and 41).

### 2.3. Antimicrobial Assay

The mean (± SD) and range for zones of inhibition (mm) of the 27-CF samples against the tested microorganisms observed were: *P. aeruginosa* 7.62 ± 6.61 (0.0–17) > *E. coli* 5.11 ± 6.29 (0.0–13) > *S. aureus* (25923) 3.03 ± 5.24 (0.0–13). None of the CF samples exhibited antimicrobial activity against *S. aureus* (MRSA) (Table 2).

Among the 27-CF samples, the largest zones of inhibition (mm ± SD) were observed for C16 against *P. aeruginosa* (17 ± 1.0), *E.coli* (12 ± 1.0.) and *S. aureus* (11 ± 1.0), C19 against *P. aeruginosa* (16 ± 1.0) and *S. aureus* (13 ± 1.0) as well as C26 against *P. aeruginosa* (14 ± 1.0), *E. coli* (13 ± 1.0), and *S. aureus* (11 ± 1.0). These three selected extracts were further studied for MIC and MBC values (µg/mL). The MIC values observed for C16, C19, and C26 against *P. aeruginosa* were 6.3, 12.5, and 12.5 with MBC values of 12.5, 25, and 25, respectively. Similar MIC (25) and MBC (50) values were observed for C16 and C19 against *E. coli* whereas, for C26 the MIC and MBC values against *E. coli* were 50 and 100, respectively (Table 3).

## 3. Statistical Analysis

The data was entered in SPSS software V 22.0 (statistical package for social science students) where PCA (principal component analysis) and K-mean cluster analysis were performed in order to evaluate the correlations and variability in GCMS as well as biological activities datasets.

### 3.1. PCA

**Figure 1:**
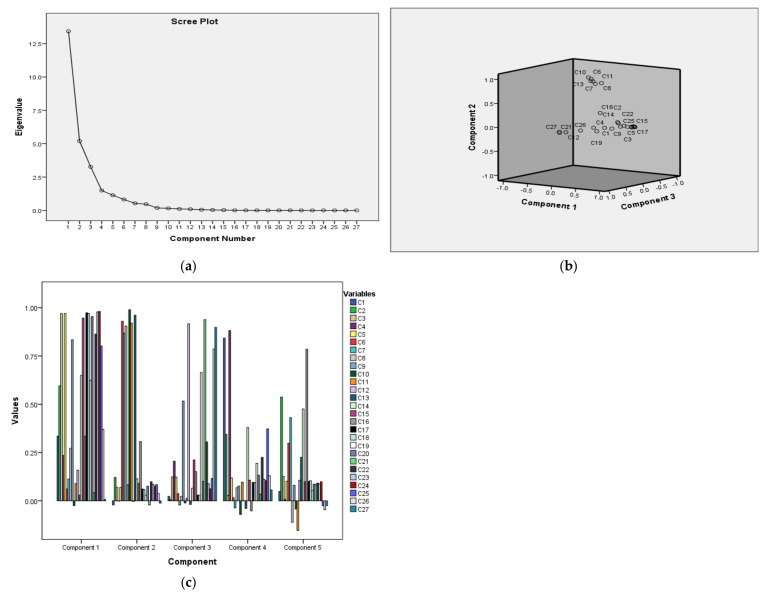
(**a**) Scree plot, (**b**) 3D view, (**c**) PCA analysis for GCMS data with components loading.

**Figure 2:**
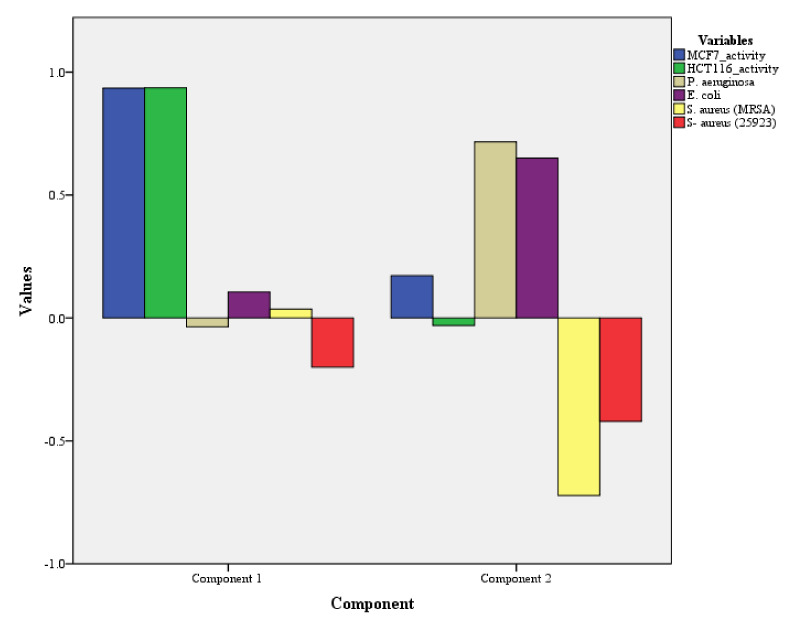
PCA analysis with components loading for cytotoxicity and antimicrobial activity.

**Figure 3:**
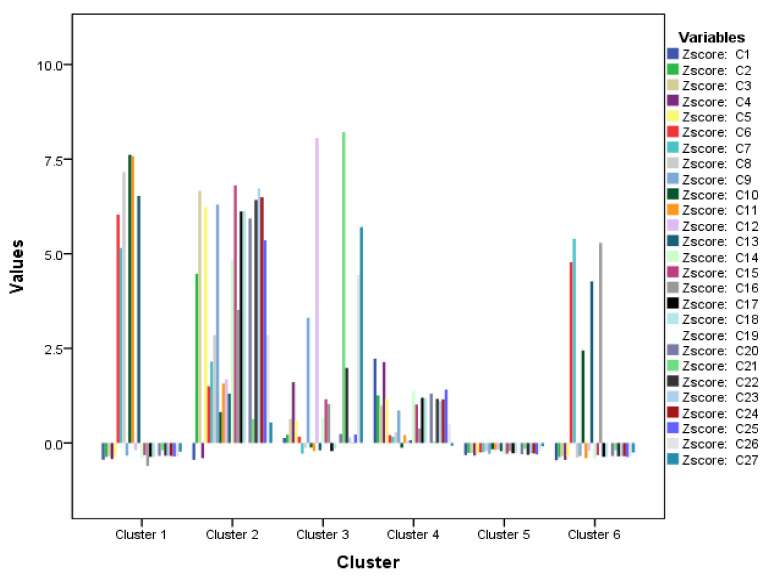
K-mean with cluster distribution for 71-volatile compounds of GCMS analysis.

Based on a specific Eigenvalue, PCA classifies the dataset into various components, representing the variability between the components and correlation among the data. For GCMS, the PCA resulted in five principal components (PC1–PC5) with a cumulative % variance of 90.806 and individual % variability of 41.78 (PC1), 19.91 (PC2), 15.30 (PC3), 7.82 (PC4), and 5.98 (PC5). The component with the highest %variability (PC1; 41.78%) consists of CF samples: C2, C3, C5, C9, C14, C15, C17–20, and C22–25, followed by PC2 19.91% which involved CF samples of C6–8, C10–11, and C13. The remaining sample was distributed in PC3–5. The CF samples with the greatest volatile components and biological activities (cytotoxicity and antimicrobial activity) i.e., C5, C13, C16, C19, and C26 are loaded in PC1 and PC2 which represent the major % cumulative variability. This suggests a strong inter-correlation for the highly variable components of PC1 and PC2 (represents the largest amount of volatile chemicals in samples loaded in these two components) as the selected extracts belong to these components. The highest cytotoxicity and antimicrobial activity for these CF samples may be attributed to the presence of large amounts of volatile components in such samples. The analysis for GCMS-PCA is supported by KMO-Bartlett’s test of Sphericity with the highest Chi-Square value of 5931.685 and a *p*-value of 0.00 with scree plots, its distribution in 3D view, and respective components are shown (Table 4, Figure 1, Figure 2 and Figure 3).

For biological activities, two components PC1 and PC2 were composed which showed a cumulative variance of 57.81% and, an individual variance of 30.07% (PC1) and 27.73% (PC2). The PCA for biological activities clearly demarcated the cytotoxicity and antimicrobial activity as seen in Table 4 and Figure 2.

The cytotoxicity was loaded in PC1 with the highest variability, followed by PC2 loaded with antimicrobial activity. The outcome of the components loading with %variability suggests a higher cytotoxicity potential for CF samples as compared to antimicrobial activity. This may be explained by the low activity against *S. aureus* (25923) and the lack of activity against *S. aureus* (MRSA). The validity of the results is supported by a high Chi-Square value of 32.19 and a *p*-value of 0.00.

### 3.2. K-Mean Analysis

The K-mean distributes a massive dataset for an experiment into various clusters based on the nearest mean of the data. The GCMS data was classified into 6-clusters, i.e., cluster 1 (1 sample), cluster 2 (1 sample), cluster 3 (1 sample), cluster 4 (9 samples), cluster 5 (58 samples), and cluster 6 (1 sample) whereas, the F- and *p*-values for each CF sample are shown in Table 5. As evident from Figure 3, cluster 2 is the more crowded cluster representing all the samples (except C1 and C4) with more amount of hydrocarbon volatile components, whereas clusters 3 (C12 and C21) and 1 (C6–8, C10, C11, and C13) represent samples with more and high amount of esters volatile components. Cluster 6 consists of the samples (C6, C7, C10, C13, and C16) with more amount of alcohol volatile compounds. For cluster 4 (9); six samples represent alcohols whereas, the remaining three samples represent the more amount of hydrocarbons volatile oils in these samples. The remaining classes for volatile components are represented in cluster 5 (58) representing the sparse distribution. The K-mean analysis successfully distributed the groups indicating a high amount of esters, alcohols, and hydrocarbons volatile components in most of the samples.

**Figure 4:**
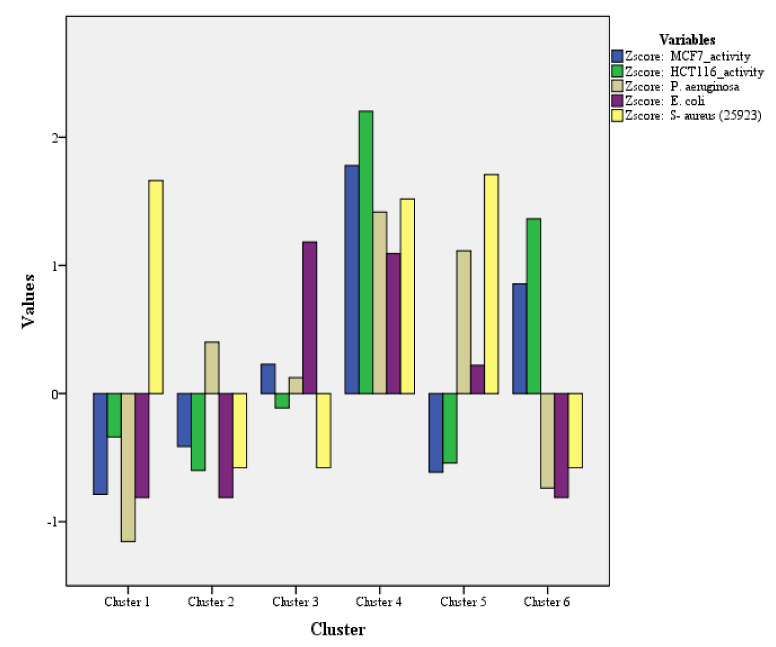
K-mean cluster analysis for cytotoxicity and antimicrobial activity of 27-CF samples.

With regard to K-mean analysis for biological activities (cytotoxicity and antimicrobial assay), high F-values (*p* ≤ 0.00) with six clusters were observed (Table 5). Cluster 4 denotes a CF sample with a significant potential role throughout the biological activities tested. This sample is represented by C16, i.e., the only sample among the 27-CF samples which exhibited significant cytotoxicity and antimicrobial activity. The next cluster representing the samples with potential for maximum activities is cluster 5. This cluster represents two samples (C19 and C26) with the highest antimicrobial activity (against *P. aeruginosa*, *E. coli*, and *S. aureus*). After cluster 5, it is cluster 6, which represents four samples. These four samples (C5, C9, C13, and C14) exhibited the highest cytotoxicity activity (against MCF7, HCT116). The remaining clusters for biological activities are shown in Figure 4. This concludes that the samples of C16 (the best among all 27-CF samples), C5, C9, C13, C14, C19, and C26 are comparatively of the best quality among the 27-different cultivars of CF.

## 4. Discussion

*Capsicum* spp. is a popular vegetable grown and consumed throughout the world [23]. More than thirty different cultivars of *Capsicum annum* L fruit (CF) have been reported [24]. The quality of CF is related to the presence of various bioactive and nutritional components [25], which are affected by several factors such as the genotype and maturity stage. Hence, the quality of CF samples may vary based on geographical origin and environmental factors, which necessitates a comprehensive research study in order to evaluate the quality variation in CF samples. The current study evaluates the quality of CF samples from twenty-seven different cultivars collected from local markets in Saudi Arabia. A method for green extraction with high yield and recovery was developed and validated for CF samples as reported [23]. The green extracts of 27-CF samples were subjected to GCMS analysis for the comparative composition of the volatile profile of each CF sample. The CF samples were then evaluated for biological activities consisting of cytotoxicity and antimicrobial, and the most potent samples were further assessed to determine the MIC and MBC of the selected samples.

The GCMS analysis revealed the presence of one hundred and nine chemical compounds from different volatile oil classes including acids, alcohols, esters, ketones, hydrocarbons, amino compounds, aldehydes, sugars, and others. The alcohols chemical class was the most dominant among the chemical classes with the major component of 1-Decanol, 2-octyl- whereas, the ester chemical class was the highest with regard to an individual occurrence where docosanoic acid, docosyl ester was found the highest amount in ester components. The order of occurrence in terms of % age for these chemical classes observed was: alcohols > hydrocarbons > esters > ketones > others > acids > sugar > aldehydes > amino compounds. The order of occurrence was confirmed by PCA and K-mean cluster analysis where the three chemical groups with widespread distribution observed in these 27-CF samples were esters (highest amount in samples), alcohols, and hydrocarbons (more distribution in samples) (*p* = 0.00). The composition of the volatile chemical constituents varies during the development stages of capsicum [26] and we found a considerable variation in the chemical composition of the 27-CF samples. It may be due to the difference in origin, exposure to various environmental factors, transport, as well as storage conditions which can affect the quality of any sample [27,28]. The predominance of alcohols and esters volatile chemical classes in our study is in-line with a similar previous report on Brazilian and other chilies [29,30].

The 27-CF samples were evaluated for their biological activities in a two-step analysis model where a general screening was performed for all the 27-CF samples at one concentration against the cell lines (MCF7, HCT116) and microorganisms (*P. aeruginosa*, *E. coli*, *S. aureus* (25923), and *S. aureus* (MRSA)). The CF samples showing the highest activities against the tested microorganisms were studied further at five different concentrations to determine the MIC and MBC values. For cytotoxicity, a range of % inhibition was observed for 27-CF samples where the three CF samples of C5 (green long serrano Holland), C13 (green long chili Saudi), and C16 (green bell pepper Saudi) exhibited the highest % inhibition. In vitro antitumor activity for CF [31], in vitro and in vivo dose-dependent apoptotic impact of capsaicin on human pancreatic cancer cells [32], as well as apoptosis and inhibition of prostate cancer cells in a mouse model [33] have been reported for CF which corroborates the results of our study. The general screening for antimicrobial activity also exhibited a wide range of zones of inhibition for 27-CF samples against *P. aeruginosa* and *E. coli* while no activity against S. aureus (MRSA) was seen for any of the 27-CF samples. The samples with the most promising antimicrobial activity were C16 (green bell pepper Saudi), C19 (red small chili Saudi), and C26 (orange small baby pepper Spain). The MIC and MBC for these selected extracts against *P. aeruginosa* revealed the lowest MIC and MBC values for C16 (green bell pepper Saudi) whereas C19 (red small chili Saudi) and C26 (orange small baby pepper Spain) showed similar MIC and MBC values. With respect to activity against *E. coli*, similar values of MIC and MBC were observed for C16 (green bell pepper Saudi) and C19 (red small chili Saudi). The C26 (orange small baby pepper Spain) CF sample showed comparatively less potential during MIC and MBC assessment against *E. coli.* Though the antimicrobial activity of CF has been mainly attributed to the presence of capsaicin and dihydrocapsaicin, it may not always be the case [34]. Therefore, further mechanistic studies are needed to confirm the antimicrobial activity of CF chemical constituents.

The statistical analysis of PCA for GCMS data showed considerable % variability for PC1 and PC2 which consisted of CF samples with a high % age of volatile chemical classes i.e., C2, C3, C5, C9, C14, C15, C17–C20, and C22–25. These samples were suggested to show a significant inter-correlation in the chemical profile of volatile oils (*p* = 0.00). The PCA for biological activities suggested a two-component loading where significant % variability was shared by the cytotoxic activity suggesting a strong cytotoxic activity for CF samples compared to antimicrobial activity. Upon further statistical analysis of the GCMS data using K-mean analysis, six clusters were observed where all the 27-CF samples were loaded in cluster 2 followed by cluster 4. The samples loaded in these clusters revealed the presence of samples under esters, alcohols, and hydrocarbons chemical classes of volatile oils. The K-mean analysis for biological activities clearly distinguished the C16 (green bell pepper Saudi) CF sample from the others, due to its unique potential in all the biological activities of cytotoxicity and antimicrobial activity. Furthermore, C19 (red small chili Saudi) and C26 (orange small baby pepper Spain) were declared the best antimicrobial CF samples whereas, C5 (green long serrano Holland), C9 (yellow capsicum Malaysia), C13 (green long chili Saudi), and C14 (red chili pepper Saudi) were observed to be the best samples for cell lines inhibition. Based on the significant overall comparative results (GCMS profile, cytotoxicity, antimicrobial activity); C16 (green bell pepper Saudi), C19 (red small chili Saudi), and C26 (orange small baby pepper Spain) were selected as samples with the best antimicrobial results and, further studied for MIC and MBC determination. The sample of C16 (green bell pepper Saudi) was declared the CF sample with the most abundant volatile constituents and highest activities in both cytotoxicity and antimicrobial assay. The PCA was observed with high Ci Square values at *p* = 0.00 whereas, the K-mean analysis exhibited high F-values with *p* ≤ 0.00 for GCMS and biological activities datasets. This study effectively highlighted the quality variation for the different cultivars of 27-CF samples based on volatile profile composition and biological activities.

## 5. Material and Methods

### 5.1. CF Samples

The 27-CF samples used in this study were collected as described in the previous study [1] which originated from different geographical origins: Holland (C1–5), Kenya (C6), Malaysia (C7–9), Morocco (C10–11), Saudi Arabia (C12–19), and Spain (C20–27). The authors found 27 different origin samples as per the availability in the local markets in the Eastern region of Saudi Arabia hence, the number of samples studied herein consisted of extracts from 27 capsicum samples.

### 5.2. Extraction of Samples

To separate the non-polar volatile components from the extracts, a non-polar and volatile solvent of n-hexane was applied in this study. The 27-CF samples (1 mg/mL) were extracted with n-hexane solvent followed by filtration (0.2 μm syringe filter), dilution (5 ppm), and volatile profile analysis via GCMS. The GCMS analysis for the volatile profiles of these 27-CF samples is reported herein.

### 5.3. GCMS Analysis

For GCMS separation, the instrument consisted of Shimadzu 2010 plus gas chromatograph with an injector (split/splitless), MS detector (QP2010), column (non-polar Rxi-5 MS capillary column; Restek Corporation) with dimensions of 30 m × 0.25 mm, 1.00 μm, whereas, Helium at a flow rate of 1.5 mL/min was used as a carrier gas. The operating condition for GC-oven consisted of: an initial temperature of 50 °C (2 min) → 150 °C (1 min) ramped at 4 °C/min → 250 °C (3 min) ramped at 8 °C/min. A temperature of 250 °C was maintained for the ion source whereas; 280 °C for the mass transfer line and mass spectra (33–450 m/z) were recorded after a 6.5 min solvent delay. For data acquisition and processing, the software used was Shimadzu GCMS Solution^®^ (version 4.52). Moreover, the area normalization process (% content) was applied for semi-quantification whereas, the NIST11 mass spectral Library database was utilized for the identification of the volatile components.

## 6. Cell Lines and Culture Used

The cell lines: human breast adenocarcinoma MCF7 (ATCC-HTB22) and human colorectal carcinoma HCT116 (ATCC-CCL247) i.e., obtained from American Type Culture Collection (ATCC) and were sub-cultured in RPMI-1640 (10% FBS). The conditions for culturing i.e., temperature (37 °C) with CO_2_ (5%), and relative humidity (100%) were properly maintained.

### 6.1. Cytotoxicity Evaluation

The 27-CF samples were subjected to an MTT assay for cytotoxicity evaluation [35,36]. Briefly, the cell lines (MCF7 and HCT116) were separately cultured (3 × 10^3^/well) in a 96-well-plate and incubated overnight (37 °C). For general cytotoxicity screening of the 27-CF samples; a single concentration of the extracts (100 μg/mL) was tested (*n* = 3; DMSO 0.1%) and the absorbance was noted with the help of a multi-plate reader. The OD (optical density) of formazan at A_550_ proportional to the number of viable cells was calculated (inhibition % age Vs control cells) Table 2. Doxorubicin (5µM) was used as a positive control for cytotoxicity evaluation.

### 6.2. Antimicrobial Activities

#### 6.2.1. Bacterial Strains and Culture Media

The strains of bacteria consisted of *Pseudomonas aeruginosa* (ATCC 15442), *Escherichia coli* (ATCC 35218), and *Staphylococcus aureus* (ATCC 25923) as well as Methicillin-resistant *Staphylococcus aureus* (MRSA) (ATCC 43300). The culture medium used for the agar-well-diffusion method was MHA i.e., Muller Hinton agar (Oxoid, CM0337) whereas, MHB i.e., Muller Hinton broth (Oxoid, CM0405) was used for the broth dilution method (MIC determination and MBC determination).

#### 6.2.2. Standard Inoculum

The selected colonies from the MHA-grown-microorganisms (37 °C; 24 h) were inoculated in MHB for a homogenous bacterial suspension formation and standardized up to 0.5 McFarland turbidity (Vitek Densichek Biomerieux analyzer).

#### 6.2.3. Agar-Well-Diffusion Method

The four bacterial strains suspended in MHB were swabbed (100 μL each) on the three directions of agar plates as per NCCLS (National Committee for Clinical Laboratory Standards) recommendations [37]. The inoculated plates were dried (10 min), and wells (6 mm) were produced with the help of sterile glass rods and filled with 27-CF samples (100 µL) individually in each well. Positive control discs (30 µg) were used for G + Ve (Amikacin) and G − Ve (Vancomycin) microorganisms whereas, DMSO (0.1% *v*/*v*) was used as vehicle control. The incubated plates (37 °C; 24 h) were examined to note the zone of inhibition (mm) for each CF sample.

#### 6.2.4. Determination of MIC and MBC

The three selected CF-extracts with the largest zones of inhibition (C16, C19, C26) were added (100 μL each) with MHB (100 μL) in a 96-well microtiter plate to make two-fold dilutions of (µg/mL) 50, 25, 12.5, 6.2 and 3.1. The 0.5 McFarland standard (10 μL) for *P. aeruginosa* or *E. coli* in MHB was poured into each CF-well of the three selected samples as well as to the positive control. The experiment was repeated in triplicate where the plates were incubated (37 °C overnight) and, the MIC and MBC were calculated as per the guidelines of the clinical and laboratory standards institute (CLSI M26-A, 1998).

### 6.3. Statistical Analysis

The dataset obtained for GCMS and biological activities was analyzed with the help of various statistical models using Statistical Package for Social Sciences software (SPSS, V 22.0). The average (± SD) was applied to simplify the data for statistical models of K-mean, PCA, and Pearson’s correlation analysis. K-mean clustering is dividing the data from a large dataset into a normalized pattern in the form of clusters whereas the clusters represent the data with more nearest mean in the dataset. The initial cluster center (iterate/classify) in combination with cluster distance was applied to distribute the data into various clusters as discussed in respective sections. For variability determination of the GCMS and biological activities datasets, the PCA model was used which is based on the Eigenvalue. The PCA provides % variability for each sample point in a dataset in terms of a positive or negative correlation where a value more ascending towards 1 shows a strong correlation among the data points. To further confirm the variability of data, Pearson’s model was applied which highlighted and confirmed the data with more correlation. The Pearson’s correlation is bivariate (positive or negative) whereas a value > 0.5 and approaching 1 is considered a strong correlation.

## 7. Conclusions

The GCMS analysis of 27-CF samples from different cultivars revealed a great variation of the chemical compounds in these samples. Seventy-one chemical compounds from various chemical groups were observed where the alcohols, hydrocarbons, and ester chemical groups were the predominant ones. The cytotoxicity assay revealed the largest % inhibition for C5 (green long serrano Holland), C9 (yellow capsicum Malaysia), C13 (green long chili Saudi), and C14 (red chili pepper Saudi) whereas, antimicrobial activity showed C19 (red small chili Saudi) and C26 (orange small baby pepper Spain) to be the most active CF samples. C16 (green bell pepper Saudi) was observed as the best quality CF sample due to its unique volatile pattern as well as the potential to inhibit the tested cell lines and microbial strains. The bioactive compounds responsible for these activities in C16 (green bell pepper Saudi) may be studied further for potential new drug development.

## Figures and Tables

**Table 1:** Cell inhibition activity of the 27 extracts against two cell lines (MTT 72 h, % ± SD μg/mL).

Sample Code	Geographical Origin	MCF7	HCT116
C1	Green long chili pepper (Holland)	9.00 ± 1.12	12.00 ± 0.45
C2	Red habanero hot pepper (Holland)	30.00 ± 4.22	12.00 ± 1.10
C3	Yellow sweet pepper (Holland)	18.00 ± 2.34	9.00 ± 0.56
C4	Green pasilla hot pepper (Holland)	25.00 ± 4.10	15.00 ± 2.0
C5	Green long serrano hot pepper (Holland)	42.00 ± 5.00	37.00 ± 1.04
C6	Red habanero hot pepper (Kenya)	40.00 ± 5.22	27.00 ± 2.31
C7	Green capsicum (Malaysia)	24.00 ± 2.00	16.00 ± 3.09
C8	Red capsicum (Malaysia)	14.00 ± 3.00	3.00 ± 0.40
C9	Yellow capsicum (Malaysia)	29.00 ± 2.22	27.00 ± 4.34
C10	Red small fresho (Morrocan)	18.00 ± 3.11	10.00 ± 1.10
C11	Orange small cayenne (Morrocan)	33.00 ± 4.41	21.00 ± 1.00
C12	Green chili pepper (Saudi)	28.00 ± 4.01	11.00 ± 1.00
C13	Green long chili pepper (Saudi)	45.00 ± 3.13	40.00 ± 2.00
C14	Red chili pepper (Saudi)	35.00 ± 5.00	27.00 ± 3.20
C15	Red bell pepper (Saudi)	39.00 ± 4.44	24.00 ± 1.10
C16	Green bell pepper (Saudi)	47.00 ± 3.00	41.00 ± 2.54
C17	Yellow bell pepper (Saudi)	29.00 ± 2.00	20.00 ± 2.00
C18	Orange bell pepper (Saudi)	24.00 ± 2.00	15.00 ± 1.22
C19	Red extra-small chili pepper (Saudi)	32.00 ± 3.00	15.00 ± 1.00
C20	Green small jalapeno chili pepper (Spain)	36.00 ± 4.33	22.00 ± 1.00
C21	Yellow small naga jolokia chili pepper (Spain)	34.00 ± 0.54	24.00 ± 2.00
C22	Red bell pepper (Spain)	33.00 ± 0.99	4.00 ± 0.45
C23	Green bell pepper (Spain)	24.00 ± 2.00	22.00 ± 2.45
C24	Yellow bell pepper (Spain)	34.00 ± 3.00	20.00 ± 3.44
C25	Red small baby pepper (Spain)	14.00 ± 2.77	11.00 ± 3.00
C26	Orange small baby pepper (Spain)	14.00 ± 5.32	13.00 ± 2.55
C27	Yellow small baby pepper (Spain)	37.00 ± 3.21	24.00 ± 2.54
Doxorubicin		85.11 ± 5.25	37.00 ± 2.01

**Table 2:** Antimicrobial activity for the 27-CF against bacterial strains.

Sample Code	Bacterial Strains				
	Geographical Origin	*P. Aeruginosa* ATCC-15442	*E. Coli* ATCC-35218	*S. Aureus (MRSA)* ATCC-43300	*S. Aureus* ATCC-25923
		Zone of Inhibition (mm ± SD)			
C1	Green long chili pepper (Holland)	R	R	R	12 ± 1.0
C2	Red habanero hot pepper (Holland)	13 ± 1.0	N.D.	R	R
C3	Yellow sweet pepper (Holland)	R	12 ± 1.0	R	R
C4	Green pasilla hot pepper (Holland)	12 ± 1.0	13 ± 1.0	R	R
C5	Green long serrano hot pepper (Holland)	R	N.D.	R	N.D.
C6	Red habanero hot pepper (Kenya)	11 ± 1.0	13 ± 1.0	R	R
C7	Green capsicum (Malaysia)	15 ± 1.0	12 ± 1.1	R	R
C8	Red capsicum (Malaysia)	12 ± 1.0	R	R	R
C9	Yellow capsicum (Malaysia)	R	R	R	R
C10	Red small fresho (Morrocan)	R	R	R	12 ± 1.0
C11	Orange small cayenne (Morrocan)	R	13 ± 1.0	R	R
C12	Green chili pepper (Saudi)	R	R	R	R
C13	Green long chili pepper (Saudi)	11 ± 1.1	R	R	R
C14	Red chili pepper (Saudi)	R	N.D.	R	N.D.
C15	Red bell pepper (Saudi)	R	13 ± 1.0	R	R
C16	Green bell pepper (Saudi)	17 ± 1.0	12 ± 1.0	R	11 ± 1.0
C17	Yellow bell pepper (Saudi)	11 ± 1.0	R	R	R
C18	Orange bell pepper (Saudi)	12 ± 1.0	R	R	R
C19	Red extra-small chili pepper (Saudi)	16 ± 1.0	N.D.	R	13 ± 1.0
C20	Green small jalapeno chili pepper (Spain)	13 ± 1.1	N.D.	R	N.D.
C21	Yellow small naga jolokia chili pepper (Spain)	12 ± 1.0	13 ± 1.0	R	R
C22	Red bell pepper (Spain)	11 ± 1.0	11 ± 1.0	R	R
C23	Green bell pepper (Spain)	R	R	R	12 ± 1.0
C24	Yellow bell pepper (Spain)	R	R	R	11 ± 1.0
C25	Red small baby pepper (Spain)	11 ± 1.1	N.D.	R	R
C26	Orange small baby pepper (Spain)	14 ± 1.0	13 ± 1.0	R	11 ± 1.0
C27	Yellow small baby pepper (Spain)	15 ± 1.0	13 ± 1.0	R	R
Amikacin		21 ± 0.00	23 ± 0.00	-	-
Vancomycin		-	-	18 ± 0.00	16 ± 0.00
DMSO		R	R	R	R

R: resistant, mm = millimeter, N.D. = not done.

**Table 3:** MIC and MBC (µg/mL) of the three selected extracts against bacterial strains.

Sample Code	Bacterial Strains				
	Geographical Origin	*P*. *Aeruginosa* (ATCC-15442)		*E. Coli* (ATCC-35218)	
		MIC	MBC	MIC	MBC
C16	Green bell pepper (Saudi)	6.3	12.5	25	50
C19	Red extra-small chili pepper (Saudi)	12.5	25	25	50
C26	Orange small baby pepper (Spain)	12.5	25	50	100

MIC: Minimum Inhibitory Concentration, MBC: Minimum Bactericidal Concentration, ATCC: American Type Culture Collection.

**Table 4:** PCA, components with respective % age, and, KMO and Bartlett’s test of Sphericity for GCMS dataset of 27-CF samples.

PCA Components for GCMS								
Components	PC1	PC2	PC3	PC4	PC5	*KMO and Bartlett’s Test*		
C1	0.335	−0.021	0.022	0.843	0.048	Kaiser–Meyer–Olkin Measure (Sampling Adequacy)		0.724
C2	0.595	0.121	0.007	0.344	0.537	Bartlett’s Test of Sphericity	Approx. Chi-Square	5931.685
C3	0.970	0.069	0.124	0.027	0.126		Df	351
C4	0.235	−0.002	0.205	0.881	0.008		Sig.	0.00
C5	0.970	0.069	0.123	0.118	0.101			
C6	0.061	0.929	0.037	0.016	0.298			
C7	0.112	0.868	−0.021	−0.037	0.431			
C8	0.271	0.905	0.023	0.068	−0.112			
C9	0.834	0.083	0.517	0.077	0.080			
C10	−0.024	0.989	−0.011	−0.071	−0.043			
C11	0.090	0.920	0.012	0.096	−0.154			
C12	0.158	−0.003	0.917	0.001	0.106			
C13	0.029	0.961	−0.019	−0.040	0.225			
C14	0.649	0.114	0.065	0.379	0.475			
C15	0.946	0.090	0.211	0.107	0.098			
C16	0.334	0.307	0.151	−0.052	0.784			
C17	0.974	0.061	0.029	0.095	0.101			
C18	0.970	0.058	0.029	0.094	0.104			
C19	0.624	0.029	0.664	0.194	0.054			
C20	0.954	0.076	0.101	0.132	0.086			
C21	0.042	−0.022	0.938	0.034	0.084			
C22	0.863	0.099	0.305	0.225	0.092			
C23	0.978	0.085	0.088	0.111	0.087			
C24	0.981	0.075	0.062	0.104	0.099			
C25	0.801	0.084	0.115	0.372	−0.025			
C26	0.370	0.037	0.786	0.130	−0.046			
C27	0.007	−0.012	0.898	0.056	−0.025			
*Individual %variance*	*41.789*	*19.910*	*15.300*	*7.824*	*5.984*			
*Cumulative %variance*	*41.789*	*61.698*	*76.999*	*84.823*	*90.806*			
**PCA for biological activities (cytotoxicity and antimicrobial assay)**								
Components	PC1	PC2				Kaiser–Meyer–Olkin Measure (Sampling Adequacy)		0.49
MCF7_activity	0.935	0.172				Bartlett’s Test of Sphericity	Approx. Chi-Square	32.19
HCT116_activity	0.936	−0.030					Df	15
*P. aeruginosa*	−0.036	0.716					Sig.	0.00
*E. coli*	0.106	0.650						
*S. aureus (MRSA)*	0.036	−0.722						
*S. aureus (25923)*	−0.200	0.421						
*Individual %variance*	*30.07*	*27.73*						
*Cumulative %variance*	*30.07*	*57.81*						

**Table 5:** K-mean cluster distribution of 27-CF samples with F- and *p*-value for GCMS and biological activities.

**K-Mean Cluster Analysis for GCMS**				
**Factors**	**F-Value**	**Significance**	**Clusters**	**Samples**
Zscore: C1	35.724	0.00	1	1
Zscore: C2	16.026	0.00	2	1
Zscore: C3	62.220	0.00	3	1
Zscore: C4	34.099	0.00	4	9
Zscore: C5	55.049	0.00	5	58
Zscore: C6	186.077	0.00	6	1
Zscore: C7	136.950	0.00	*Total*	*71*
Zscore: C8	111.805	0.00		
Zscore: C9	104.855	0.00		
Zscore: C10	236.243	0.00		
Zscore: C11	105.068	0.00		
Zscore: C12	1775.197	0.00		
Zscore: C13	181.475	0.00		
Zscore: C14	25.597	0.00		
Zscore: C15	99.164	0.00		
Zscore: C16	24.569	0.00		
Zscore: C17	47.512	0.00		
Zscore: C18	47.646	0.00		
Zscore: C19	36.021	0.00		
Zscore: C20	51.863	0.00		
Zscore: C21	1136.871	0.00		
Zscore: C22	124.635	0.00		
Zscore: C23	87.037	0.00		
Zscore: C24	68.361	0.00		
Zscore: C25	38.055	0.00		
Zscore: C26	11.216	0.00		
Zscore: C27	11.902	0.00		
**Cluster Analysis for Cytotoxicity and Antimicrobial Assay**				
**Factors**	**F-value**	**Significance**	**Clusters**	**Samples**
Zscore: MCF7_activity	3.07	0.031	1	4
Zscore: HCT116_activity	6.68	0.001	2	7
Zscore: *P. aeruginosa*	4.36	0.007	3	9
Zscore: *E. coli*	44.59	0.000	4	1
Zscore: *S. aureus* (25923)	1087.74	0.000	5	2
			6	4
			*Total*	*27*

## Data Availability

Not Applicable.

## Supplementary Materials

The following supporting information can be downloaded at: https://www.mdpi.com/article/10.3390/plants11081022/s1, Table S1: GCMS data for the samples, Figure S1: representative GCMS chromatograms for the samples.
